# Japanese medical researchers’ perceptions of quantitative research evaluation metrics and their psychological well-being: a cross-sectional study

**DOI:** 10.1265/ehpm.25-00194

**Published:** 2025-09-20

**Authors:** Akira Minoura, Keisuke Kuwahara, Yuhei Shimada, Hiroko Fukushima, Makoto Kondo, Takehiro Sugiyama

**Affiliations:** 1 Department of Hygiene, Public Health and Preventive Medicine, Showa Medical University School of Medicine, Shinagawa-ku, Japan; 2 Department of Epidemiology and Prevention, Center for Clinical Sciences, Japan Institute for Health Security, Shinjuku-ku, Japan; 3 Department of Public Health, Yokohama City University School of Medicine, Yokohama, Japan; 4 Department of Health Data Science, Graduate School of Data Science, Yokohama City University, Yokohama, Japan; 5 Graduate Schools for Law and Politics, The University of Tokyo, Bunkyo-ku, Japan; 6 Diabetes and Metabolism Information Center, National Institute of Global Health and Medicine, Japan Institute for Health Security, Shinjuku-ku, Japan; 7 Department of Pediatrics, University of Tsukuba Hospital, Tsukuba, Japan; 8 Department of Child Health, Institute of Medicine, University of Tsukuba, Tsukuba, Japan; 9 Department of Anatomy and Neuroscience, Graduate School of Medicine, Osaka Metropolitan University, Osaka, Japan; 10 Department of Health Services Research, Institute of Medicine, University of Tsukuba, Tsukuba, Japan

**Keywords:** Medical researcher, Research evaluation, Psychological well-being, Japanese, Clinical medicine, Basic medicine, Social medicine

## Abstract

**Background:**

Supporting the mental health of researchers is essential to maintaining human resources and advancing science. This study investigated the association between Japanese medical researchers’ perceptions of research evaluation processes and their psychological well-being.

**Methods:**

We performed a web-based self-administered questionnaire survey. The questionnaires were distributed to each academic society through the Japanese Association of Medical Sciences from December 2022 to January 2023. These questionnaires targeted medical researchers. Exposure was the medical researchers’ perceptions of quantitative indicators for evaluating medical research and researchers. The outcome was psychological well-being, measured using the Japanese version of the World Health Organization-Five Well-Being Index (WHO-5). Multivariable-adjusted logistic regressions were conducted to investigate the association between individual attitudes toward research evaluation and psychological well-being. Stratified analyses by research fields, i.e., clinical, basic, and social medicine, were also performed.

**Results:**

A total of 3,139 valid responses were collected. After excluding 176 responses from research fields of other than clinical, basic, or social medicine, 2,963 researchers (2,185 male, 737 female, and 41 other) were analyzed. Prevalence of poor well-being (WHO-5 score <13) was 28.3% in the researchers. The highest number of medical researchers was in clinical medicine (n = 500) followed by basic medicine (n = 217) and social medicine (n = 121). Medical researchers who considered research funding slightly important/not important for researcher evaluation had poorer psychological well-being than those who considered it especially important (slightly important: adjusted odds ratio (aOR) 1.33, 95% confidence interval (CI) 1.03–1.71; not important: aOR 1.53, 95%CI 1.10–2.12). This tendency was stronger among basic medical researchers than clinical or social medical researchers. The research field significantly modified the relationship between research funding received and interaction with poor psychological well-being both additively (P = 0.030) and multiplicatively (P = 0.024).

**Conclusions:**

The discrepancy between medical researchers’ attitudes toward research evaluation and the current state of research evaluation in their research community may worsen their psychological well-being. The influence of this discrepancy differs among clinical, basic, and social medicine. Appropriate evaluation of medical research and researchers in each field can facilitate improving their psychological well-being via the resolution of this discrepancy.

**Supplementary information:**

The online version contains supplementary material available at https://doi.org/10.1265/ehpm.25-00194.

## Graphical Abstract

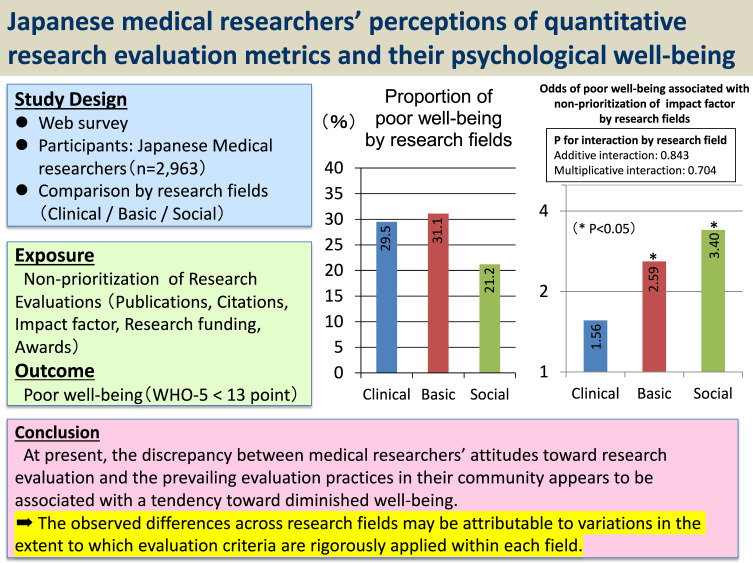

## Background

Optimal psychological well-being and mental health in the workplace are critical for work performance [1, 2]. Researchers suffer from psychological problems, such as distress and mental ill-health, and measures to mitigate such psychological problems are essential not only for researchers’ well-being but also for the development of science [3]. Recently, psychological concerns in academia, such as stress, burnout, anxiety, and depression, have increased among faculty [4, 5]. Among various types of researchers, physician researchers often face unique challenges due to the dual demands of clinical and academic responsibilities. According to a previous study in Japan, around 20% of early-career physician researchers reported engaging in negotiations, most commonly to reduce clinical duties. These negotiations were associated with spending no more than 18 hours per week on research [6]. The challenging work environment in which researchers operate, the need to balance their personal and professional responsibilities, and the constant pressure they face to increase their research productivity while juggling multiple responsibilities, including teaching, research, mentoring, professional development, and social activities, all impact their mental health and overall psychological well-being [7]. Therefore, an enhancement of the organizational climate, particularly for researchers experiencing high levels of psychological distress, is necessary to prevent them from experiencing psychological problems [8]. A holistic approach, encompassing individual skills and organizational efforts, is urgently needed to safeguard researchers’ psychological well-being and mental health.

Workplace evaluations affect workers’ mental health, including researchers [9]. Researchers’ evaluations include qualitative and quantitative aspects. Unlike internal workplace evaluations, research evaluation metrics are, at least superficially, external and exchangeable. The aphorism “Publish or Perish” supports the general belief that, in the research community, a researcher’s publication count decides whether they are highly esteemed or not [10]. However, quantitative metrics like article counts and citations may not fully capture the true nature of scientific discovery [11].

High ratings based on quantitative indicators are often unattainable, which may affect the health of those rated poorly [12]. Among people who do not agree with the way research is evaluated, the gap itself or poor evaluation received can cause psychological stress. Our previous study using the same dataset reported that Japanese medical researchers prioritized research funding and the number of papers published in English when evaluating their peers, and that perceptions of appropriate evaluation varied by research field [13]. Accordingly, we hypothesized that the association between research evaluation and assessment processes and the psychological well-being of researchers may differ depending on the research field. However, no study has examined the relationship between research/researcher evaluation processes and the psychological well-being of researchers. Therefore, this study investigated the relationship between medical researchers’ perceptions of research evaluation metrics and their psychological well-being.

## Methods

### Study design and setting

We designed our survey as U40 (Under 40 years as of 2020) members from the Scientific Committee for the 31st General Assembly of the Japanese Association of Medical Sciences. This study was a analysis of cross-sectional survey data collected from medical researchers in Japan between December 14, 2022, and January 17, 2023. Although this study was designed to survey medical researchers’ awareness of research evaluation, a secondary objective was to develop a self-reported questionnaire on different research evaluation processes and researchers’ psychological well-being. The survey process details are in our previous study [13]. The questionnaire was administered via a web survey to all participants. Medical researchers vary widely in their research evaluation axes across research fields. Therefore, associations between research ratings and poor well-being were shown separately for clinical, basic, and social medicine.

### Study participants

**Fig. 1:**
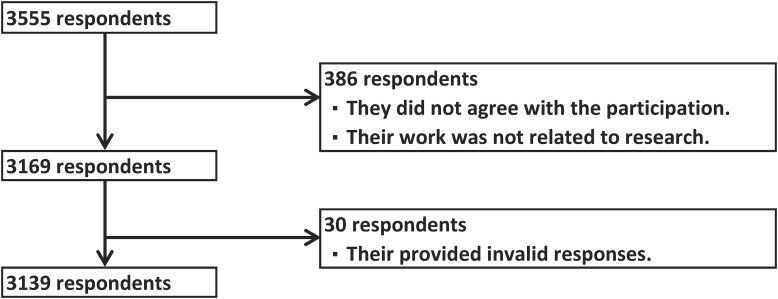
Flowchart of study participant selection

On our survey website, eligible participants were notified about the survey website and asked for their consent to participate. At the beginning of our questionnaire, a question was asked whether “I consent to the survey,” or “I do not consent to the survey, or my work does not involve research.” Only those who answered “I consent to the survey” were allowed to answer the subsequent questions. If eligible participants took maternity leave or other time off, they were instructed to answer work-related questions before taking the leave or the time off. Additionally, we excluded those who provided invalid responses by giving the same answer to all questions due to the questionable validity of their answers. Flowchart of study participant selection shows in Fig. 1.

### Study procedures

#### Exposure variable

The main exposure variable was medical researchers’ perceptions of quantitative indicators used for research evaluation. The participants were asked to assess the importance of six quantitative indicators when evaluating their peers on a 5-point scale ranging from 1 to 5, with 1 indicating “Especially important,” 2 “Important,” 3 “Slightly important,” 4 “Not important,” and 5 “I do not know.” The six indicators included “The number of papers a researcher has published in English-language journals,” “The number of papers a researcher has published in Japanese-language journals,” “The number of citations a researcher’s publications has,” “The impact factor (IF) of a journal where a researcher has published his work,” “Research funding received by a researcher,” and “Awards received by a researcher.”

#### Outcome variable

The outcome variable was psychological well-being, assessed using the World Health Organization-Five Well-Being Index (WHO-5) [14]. The WHO-5 has been translated into over 30 languages and used in research projects worldwide, and this index makes it possible to measure positive psychological health, such as vitality and positive mood. The WHO-5 measures positive aspects of mental health, such as vitality, positive mood, and overall emotional well-being. It has been translated into over 30 languages and is widely used in international research. Each item is rated on a 6-point Likert scale ranging from 0 to 5, where 0 indicates “not at all,” 1 indicates “some of the time,” 2 indicates “less than half the time,” 3 indicates “more than half the time,” 4 indicates “most of the time,” and 5 indicates “all of the time.” The total score, calculated by summing the five-item scores, ranges from 0 to 25, with 0 indicating the worst possible quality of life and 25 indicating the best possible quality of life. A score of ≤12 points indicates psychological well-being and potential depression, whereas a score of >12 points indicates better psychological well-being [14].

#### Covariates

Age (Below 20, 20–24, 25–29, 30–34, 35–39, 40–44, 45–49, 50–54, 55–59, 60–64, 65–69, 70 and above, or I do not wish to answer), gender (female, male, other, or I do not wish to answer), marital status (single, married, bereaved, divorced, or I do not want to answer, other), and research field (clinical medicine, basic medicine, and social medicine), were collected through the questionnaire as the multiple answers. Because of the similarity of the answer pattern, those who answered two or more research fields were classified into one of them. For example, all those who selected only basic medicine into “Basic”, and all those who selected only social medicine into “Social”. The details of the classification of research fields and the covariates were described in our previous study [13].

### Statistical analyses

In the descriptive analysis, the proportion of all researchers and researchers in each field was described by gender, age group (<40, 40s, 50s, 50s<), marital status, WHO-5 score, and perceptions of quantitative research evaluations. The research field described associations between perceptions of research evaluations and psychological well-being among researchers. The Cochran–Armitage test assessed linear trends in perceptions of research evaluations and well-being based on the research field. A multivariable-adjusted logistic model was used to describe the association between researchers’ characteristics and perceptions of research evaluations and poor well-being, and we performed both aggregated models and separated models for research fields. Since the characteristics of quantitative indicators differ to the research fields, we investigated additive/multiplicative interactions between perceptions of research evaluations and the research field on psychological well-being. Additive interaction was assessed by comparing risk differences, while multiplicative interaction was examined using interaction terms in logistic regression models [15]. All statistical analyses were performed using JMP Pro 17.0 (SAS Institute Inc. Cary, NC, USA). In this study, P-values less than 0.05 (two-sided) were defined statistically significant. The Strengthening the Reporting of Observational Studies in Epidemiology (STROBE) guidelines checklist has been detailed in the online supplemental material.

### Ethical considerations

The study protocol was approved by the Institutional Review Board of the National Center for Global Health and Medicine (NCGM-S-004530-01).

## Results

### Participant selection

A total of 3,139 valid responses were collected. After excluding 176 responses stating that their field of research was neither clinical, basic, nor social medicine, 2,963 researchers (2,185 males, 737 females, and 41 others) were included in the analysis.

### Participant characteristics

Table 1 shows the characteristics of medical researchers by research field. The largest number of researchers were male (73.4%) in their 40s (30.7%) and 50s (27.5%). Of the 2,963 researchers, 1,696 were in clinical medicine, 697 in basic medicine, and 570 in social medicine. The WHO-5 scores of 28.3% of the researchers were rated poor psychological well-being.

**Table 1:** Characteristics of medical researchers by research fields (Clinical, Basic and Social).

		**Overall** **(n = 2963)**	**Clinical** **(n = 1696)**	**Basic** **(n = 697)**	**Social** **(n = 570)**
**Gender**					
	**Female**	737 (24.9)	371 (21.9)	143 (20.5)	223 (39.1)
	**Male**	2185 (73.7)	1298 (76.5)	543 (77.9)	344 (60.4)
	**Others**	41 (1.4)	27 (1.6)	11 (1.6)	3 (0.5)
**Age**					
	**<40**	619 (20.9)	312 (18.4)	171 (24.5)	136 (23.9)
	**40s**	909 (30.7)	508 (30.0)	222 (31.9)	179 (31.4)
	**50s**	814 (27.5)	496 (29.3)	175 (25.1)	143 (25.1)
	**50s<**	578 (19.5)	357 (21.1)	114 (16.4)	107 (18.8)
**Marriage**					
	**Single**	380 (12.8)	182 (10.7)	105 (15.1)	93 (16.3)
	**Married**	2389 (80.6)	1408 (83.0)	546 (78.3)	435 (76.3)
	**Others**	194 (6.6)	106 (6.3)	46 (6.6)	42 (7.4)
**WHO-5 score**					
(0–25)		15 (12,19)	15 (12,19)	15 (11,19)	16 (13,20)
**Lower psychological well-being**					
(WHO-5 score <13)		838 (28.3)	500 (29.5)	217 (31.1)	121 (21.2)

Expect where indicated, n (%), values are medians (25^th^ and 75^th^ percentiles).

For each characteristic, the rows total 100%.

### Characteristics of participants in different research fields

The highest proportion of male researchers was in the basic medicine field (77.9%), followed by the clinical medicine (76.5%) and social medicine fields (60.4%). A similar trend was observed in the proportion of researchers with poor psychological well-being. The highest proportion of medical researchers with poor psychological well-being was found in the basic medicine field (31.1%), followed by the clinical medicine (29.5%) and social medicine fields (21.2%).

### Relationship between researchers’ perceptions of research evaluation metrics and their psychological well-being

Table 2 shows the associations between research ratings and poor well-being, separately for clinical, basic, and social medicine. The number of English-language articles was not a factor in evaluating researchers in clinical, basic, or social medicine fields who tended to have poor psychological well-being. Researchers in clinical, basic, or social medicine who did not focus on the number of English-language articles tended to have poor psychological well-being. Conversely, no association was observed between focusing on the number of Japanese-language articles and poor psychological well-being. The lack of focus on the IF tended to be associated with the poor psychological well-being of researchers in clinical, basic, or social medicine fields. The lack of focus on research funding was associated with the poor psychological well-being of researchers in clinical or basic medicine fields. The lack of focus on research awards was associated with the poor psychological well-being of researchers in the clinical medicine field.

**Table 2:** Associations between research evaluations and lower psychological well-being^1)^ among medical researchers by research field.

	**Research** **fields**	**Especially** **important**	**Important**	**Slightly** **important**	**Not** **important**	**Trend P^2)^**
**The number of papers published ** **in English-language journals**	**Clinical**	144 (27.2)	275 (29.7)	68 (34.3)	10 (29.4)	0.048
	**Basic**	66 (24.9)	112 (33.0)	33 (44.0)	6 (40.0)	0.002
	**Social**	35 (18.0)	54 (19.4)	27 (34.6)	4 (28.6)	0.006

**The number of papers published ** **in Japanese-language journals**	**Clinical**	16 (31.4)	168 (29.7)	229 (31.1)	84 (25.0)	0.123
	**Basic**	2 (18.6)	28 (25.9)	93 (30.4)	93 (34.2)	0.065
	**Social**	5 (20.0)	44 (23.9)	45 (18.4)	26 (23.0)	0.402

**The number of citations**	**Clinical**	76 (27.5)	209 (28.2)	152 (31.2)	52 (31.3)	0.043
	**Basic**	49 (27.8)	88 (28.6)	58 (37.7)	21 (40.4)	0.025
	**Social**	11 (11.6)	44 (18.3)	45 (27.6)	19 (32.8)	<0.001

**Impact factors**	**Clinical**	83 (25.0)	246 (29.0)	131 (32.7)	33 (34.0)	0.004
	**Basic**	46 (25.7)	101 (31.1)	54 (35.1)	15 (44.1)	0.014
	**Social**	8 (11.4)	49 (19.3)	43 (24.7)	20 (33.3)	0.005

**Research funding received**	**Clinical**	79 (26.4)	224 (30.0)	123 (28.5)	54 (30.9)	0.046
	**Basic**	47 (25.7)	105 (29.9)	48 (38.7)	17 (47.2)	0.003
	**Social**	18 (19.2)	51 (18.2)	37 (27.6)	14 (29.2)	0.076

**Awards received**	**Clinical**	40 (25.6)	172 (27.7)	195 (30.3)	79 (32.6)	**0.010**
	**Basic**	21 (30.4)	66 (27.4)	88 (32.4)	40 (37.4)	0.153
	**Social**	8 (21.1)	26 (16.1)	62 (23.1)	24 (27.0)	0.116

n (%)

%: Percentage of researchers shows lower psychological well-being for each answer.

1) Lower psychological well-being defined as WHO-5 scores (0–25) less than 13.

2) Trend P were calculated using the Cochran-Armitage test.

### The relationship between researchers’ perceptions of research evaluations and poor psychological well-being among all researchers

The researchers’ perceptions of the number of papers published in Japanese-language journals and the number of awards received by researchers had no impact on their psychological well-being.

### The relationship between researchers’ perceptions of research evaluations and poor psychological well-being according to the research field

Table 3 shows the association between individual attitudes toward research evaluation and well-being among medical researchers adjusting for gender, age, and marital status. The results of the crude model are shown in Appendix Table 1. Medical researchers who reported that the number of papers published in English-language journals was slightly important/important (adjusted odds ratio (aOR): 1.23, 95% confidence interval (CI): 1.02–1.48/aOR: 1.71, 95%CI: 1.31–2.24) had poor well-being than those who reported it was especially important.

**Table 3:** Association between individual attitudes toward research evaluation and lower psychological well-being (Adjusted model).

	**Overall** **(n = 2963)**	**Clinical** **medicine** **(n = 1696)**	**Basic** **medicine** **(n = 697)**	**Social** **medicine** **(n = 570)**	**Additive** **interaction**	**Multiplicative** **interaction**
	**[aOR]**	**[aOR]**	**[aOR]**	**[aOR]**	**[p-value]**	**[p-value]**
**The number of papers published** **in English-language journals**						
** Especially important**	Ref.	Ref.	Ref.	Ref.	0.497	0.399
** Important**	1.23 (1.02–1.48)	1.15 (0.90–1.46)	1.55 (1.06–2.25)	1.05 (0.64–1.72)		
** Slightly important**	1.71 (1.31–2.24)	1.37 (0.95–1.95)	2.54 (1.44–4.46)	2.36 (1.26–4.43)		
** Not important**	1.43 (0.82–2.51)	1.21 (0.56–2.64)	2.47 (0.81–7.52)	1.84 (1.26–4.43)		
**The number of papers published** **in Japanese-language journals**						
** Especially important**	Ref.	Ref.	Ref.	Ref.	0.354	0.996
** Important**	1.00 (0.60–1.68)	0.91 (0.48–1.72)	0.86 (0.15–4.95)	1.21 (0.42–3.53)		
** Slightly important**	1.01 (0.61–1.67)	0.97 (0.51–1.81)	1.08 (0.20–5.97)	0.79 (0.27–2.30)		
** Not important**	1.00 (0.59–1.68)	0.73 (0.38–1.41)	1.45 (0.26–7.98)	1.06 (0.35–3.23)		
**The number of citations**						
** Especially important**	Ref.	Ref.	Ref.	Ref.	0.254	0.142
** Important**	1.09 (0.86–1.38)	1.07 (0.78–1.47)	0.93 (0.61–1.44)	1.68 (0.82–3.47)		
** Slightly important**	1.34 (1.04–1.72)	1.18 (0.85–1.65)	1.44 (0.88–2.34)	2.73 (1.31–5.70)		
** Not important**	1.48 (1.07–2.04)	1.19 (0.78–1.83)	1.69 (0.86–3.30)	3.48 (1.46–8.32)		
**Impact factors**						
** Especially important**	Ref.	Ref.	Ref.	Ref.	0.843	0.704
** Important**	1.27 (1.01–1.59)	1.24 (0.92–1.66)	1.34 (0.88–2.05)	1.85 (0.82–4.20)		
** Slightly important**	1.49 (1.16–1.92)	1.47 (1.05–2.04)	1.66 (1.02–2.73)	2.42 (1.05–5.57)		
** Not important**	1.82 (1.27–2.61)	1.56 (0.95–2.56)	2.59 (1.19–5.67)	3.40 (1.33–8.70)		
**Research funding received**						
** Especially important**	Ref.	Ref.	Ref.	Ref.	0.030	0.024
** Important**	1.18 (0.94–1.48)	1.25 (0.92–1.70)	1.35 (0.88–2.06)	0.86 (0.46–1.60)		
** Slightly important**	1.33 (1.03–1.71)	1.12 (0.80–1.58)	2.10 (1.25–3.53)	1.50 (0.77–2.91)		
** Not important**	1.53 (1.10–2.12)	1.26 (0.82–1.92)	3.52 (1.60–7.72)	1.69 (0.72–3.93)		
**Awards received**						
** Especially important**	Ref.	Ref.	Ref.	Ref.	0.713	0.608
** Important**	1.00 (0.73–1.37)	1.16 (0.77–1.75)	0.83 (0.45–1.52)	0.65 (0.26–1.63)		
** Slightly important**	1.17 (0.86–1.59)	1.25 (0.84–1.88)	1.19 (0.66–2.15)	1.06 (0.44–2.51)		
** Not important**	1.36 (0.96–1.93)	1.43 (0.90–2.27)	1.37 (0.70–2.70)	1.20 (0.46–3.11)		

aOR (95% confidence interval)

aOR: odds ratio adjusted for gender, age and marriage.

However, when medical researchers were divided into three categories: clinical, basic, and social medicine, this tendency was significant in the basic medicine field (aOR: 1.55, 95%CI: 1.06–2.25/aOR: 2.54, 95%CI: 1.44–4.46). No significant association was observed in clinical medicine. Medical researchers who reported that the research funding received was slightly important/important (aOR: 1.33, 95%CI: 1.03–1.71/aOR: 1.53, 95%CI: 1.10–2.12) had poorer well-being than those who reported that it was especially important.

### Heterogeneity of relationship between the researchers’ perceptions of evaluation and psychological well-being by research fields

After being divided into three categories, addictive/multiplicative interactions were found to be significant. This tendency was significant only in basic medicine (aOR: 2.10, 95%CI: 1.25–3.53/aOR: 3.52, 95%CI: 1.60–7.72). Associations were observed between the number of citations, IF, research funding received, and psychological well-being. Particularly, an association was observed between IF and the well-being of researchers in the clinical, basic, or social medicine fields. No association was observed between the awards received and the well-being of medical researchers. Furthermore, no association was observed between the number of papers published in Japanese-language journals and well-being. Without research funding, addictive/multiplicative interactions between research evaluations and research fields on poor well-being were not significant.

## Discussion

Medical researchers who did not prioritize metrics of research evaluation tended to have poorer psychological well-being than those who did, based on the following quantitative indicators: the number of papers published in English-language journals, the number of citations, IF, and research funding received. The association between the attitude toward quantitative indicators and poor psychological well-being varied by research field. The positive associations of research funding received with poor psychological well-being were more strengthened in basic medicine. The positive associations of the number of citations with poor psychological well-being were more strengthened in social medicine. To our knowledge, this is the first study to demonstrate that medical researchers’ perceptions of research evaluation would influence their psychological well-being.

### The focus on quantitative indicators influences researchers’ psychological well-being

Our results showed that medical researchers with a discrepancy between perceived and actual research evaluation tend to exhibit poor psychological well-being. Researchers who did not place importance on the number of papers published in English-language journals in research evaluation had poor psychological well-being. This finding seems in line with academic culture at least in Japan. Although we did not find any observational studies, the detrimental effects of contemporary publication culture are also frequently mentioned in other countries [16]. These effects are often focused on citation measures (like the IF and H-index), competition, the funding system, and publication bias among researchers. English-language journals tend to have higher IF than Japanese-language journals, creating a bias toward research published in English. In academic communities, the number of papers published in English-language journals, the number of citations, and IF are often highly evaluated by funders and institutional managers when determining funding allocation or researcher recruitment in universities [17]. Whereas some researchers internalize these values and aim to write many English papers with high IFs, others cannot accept these values and are confused by the gap between the evaluation criteria of those around them and their values. Against this background, dissatisfaction with research evaluations based on quantitative indicators may be associated with poor psychological well-being among researchers.

Moreover, our results suggest that the psychological well-being of medical researchers who do not prioritize research funding is poor. Many institutions in Japan use quantitative indicators as a criterion for evaluating researchers, at least in part because they can obtain overhead costs and supplement their own research funds, which are becoming increasingly scarce. Funding agencies may use previous funding history to confirm that the applying researcher is competitive. Researchers who find it unacceptable to be evaluated on the level of research funding rather than on their research itself may experience poor psychological well-being. Although the use of quantitative indicators has been met with significant international skepticism, their application remains particularly widespread in Japan. Japan currently suffers from abuses of quantitative indicators, which is problematic due to its significant departure from the Declaration on Research Assessment and the Leiden Manifesto. The present study highlights another detrimental effect of overreliance on quantitative indicators and may contribute to the growing body of criticism. Further examination of the limitations of the quantitative indicators from this cross-sectional survey may reinforce warnings against their overreliance.

### The relationship between perceptions of research evaluation and psychological well-being by research field

Another interesting finding of the present study is that the relationship between each quantitative indicator and psychological well-being varied depending on the research field. This study observed associations between quantitative indicators for research evaluations and psychological disorders in basic and social medicine but hardly in clinical medicine. In basic medicine, the number of papers published in English-language journals, IF, and research funding received were associated with psychological well-being. By contrast, in social medicine, the number of papers published in English-language journals, the number of citations, and IF were associated with psychological well-being. In clinical medicine, however, only IF was associated with psychological well-being. Particularly for researchers in basic and social medicine, the way research is evaluated and researchers’ attitudes toward it may be an important factor in addressing psychological well-being. These differences by research field may be because of following reasons. For the research evaluations by research field, social medicine also ranks based on social activities, but basic medicine relies highly dependent on research evaluations [18].

In comparison, the way physicians are evaluated may vary depending on how much effort they put into research; it is possible that researchers’ evaluations may differ by whether they provide clinical services. In our results, differences in research fields may confirm the importance from the perspective of medical researchers’ prevention of psychological disorders. The increasing number of researchers and the development of information and communication technology have promoted the diversification of research titles and increased the number of articles from year to year [18]. There is concern that evaluating research based solely on quantitative indicators may not adequately assess the originality and novelty of the research. The diversification of research is essential for developing science, evaluating research and researchers challenging. Researchers who feel that quantitative indicators undervalue the originality and novelty of their research may experience poor psychological well-being. The acceptance of pluralistic values by research field may serve as a moderating factor in the relationship between perceptions of research evaluation and the psychological well-being of medical researchers.

The results of this study also suggest gender difference between researchers’ perceptions of quantitative research evaluation metrics and their psychological well-being. In basic medicine, where the proportion of females is the lowest, not placing importance on research funding received may be related to poor psychological well-being. Previous studies on researchers in biology have shown that female researchers publish fewer articles, and their names do not feature as much as those of male researchers among the lists of coauthors, possibly due to fewer opportunities for female researchers to interact with other researchers [4, 19]. Diversity in researchers’ evaluation methods allows appropriate evaluation of each researcher’s efforts and can prevent poor psychological well-being. For the research evaluations, social medicine also ranks based on social activities, but basic medicine relies highly dependent on research evaluations [20]. In academia, bias toward research evaluation has become a problem [20]. The acceptance of pluralistic values may serve as a moderating factor in the relationship between perceptions of research evaluation and the psychological well-being of medical researchers. Measures to improve the situation should be considered, particularly in basic and social medicine.

### Researchers’ attitudes toward research funding received and their psychological well-being

The findings of this study suggested that, particularly in basic medicine, not prioritizing the acquisition of research funding in researchers’ evaluation was associated with poorer psychological well-being. We observed a significant difference in the association between perceptions about funding and psychological well-being by research field. Tsugawa et al. reported that participation in large-scale research projects increases the likelihood of securing research funding, particularly in basic medicine [21]. Furthermore, maintaining experimental environments—including animals, reagents, and equipment—incurs substantial costs in basic medicine, making the acquisition of research funding essential for the continuity of research activities. Some researchers may prioritize the ease of obtaining research funding over academic interest or their own sense of importance when selecting research topics in order to continue their research. In this context, those who actually recognize that funding received is not important for their evaluation may be susceptible to psychological distress when faced with pressure to obtain funding. The discrepancy between their values and what they should do may damage self-esteem. Originally, funding allocation policies are determined with the aim of maximizing research results.

On the other hand, it is worth noting that such discrepancy or concerns about funding shortages may cause mental health issues among researchers, which may lead to a shortage of research personnel and a decline in the attractiveness of the research field. Considering that perceptions of research evaluation may vary by research field, age, and other factors, it is important to include researchers from diverse backgrounds in discussions regarding funding allocation. One potential solution is to implement smaller, widely distributed funding schemes in academia, which may help sustain research activities by preventing researchers from becoming inactive due to funding discontinuity [22].

### Strengths and limitations

This study is the first to examine the association between research evaluations and psychological well-being among Japanese medical researchers by clinical, basic, and social stratification. The findings of this study are expected to improve researchers’ evaluation methods and, indirectly, their research performance. This study has some limitations. First, using a web-based self-administered survey could lead to selection bias, as researchers who frequently check the internet or email and are used to answering a web-based questionnaire may have been more likely to respond to the survey. Second, the survey was conducted during the COVID-19 pandemic, which might have affected the researchers’ psychological well-being. Physical and social distancing measures during COVID-19 have a contemporaneous impact on health and psychological well-being among young people [23]. As the pandemic evolves, it is essential to consider how to mitigate any longer-term health impacts of physical distancing restrictions among medical researchers. Third, this was a cross-sectional study. Although this study showed an association between research evaluations and psychological well-being among medical researchers, causal inferences cannot be drawn between researchers’ perceptions of research evaluations and their psychological well-being. Fourth, this study cannot conduct an age-specific analysis because the participants’ ages are skewed toward seniors [13]. The career progression of medical researchers is essential for the development of medicine because it places the right people in the right positions. In Japan, young researchers (with a Ph.D. less than 8 years ago) are typically early career scientists who have recently completed their PhDs and are pursuing postdoctoral research or other research positions [21]. They may receive fellowships or other support to focus on their research topics, and the Japanese government has encouraged young scientists to consider careers in industry, as academic jobs are scarce [24]. Conversely, senior researchers are more established in their careers and hold permanent academic positions. A previous study analyzed the impact of supervisors’ research style on young biomedical researchers in Japan and showed the challenges medical schools face in developing research activities for innovation [25]. Additionally, gender differences have not been adequately examined. Although over 70% of the respondents were male, this gender distribution is consistent with that of previous studies of Japanese medical researchers [26]. Further longitudinal studies are needed to investigate these relationships. Finally, some researchers belong to multiple research fields and institutions, which makes interpreting the research environment and effects for reseacher’s health difficult. Moreover, surveys targeting Japanese medical researchers tend to have a response rate of around 20%, so our results must be interpreted with caution. For example, the response rate to a survey of Japanese Surgical Society members was just over 25%, indicating that many researchers with little interest in the survey content may not have responded [27]. In our survey, researchers with a strong interest in research evaluation may have been overrepresented, resulting in underrepresentation of neutral or indifferent opinions regarding research evaluation. Therefore, establishing a systematic registry to monitor and evaluate researchers’ well-being should be considered a priority. In the future research, it will be important to categorize researcher’s properties more specifically to improve our understanding of the environment surrounding researchers.

## Conclusions

This study showed that medical researchers’ attitudes toward research evaluation can affect their psychological well-being. A discrepancy between medical researchers’ attitudes toward research evaluation and the current state of research evaluation in the research community to which they belong may deteriorate their psychological well-being. In addition, the relationship between attitudes towards research evaluation and psychological well-being may differ by the research field. Further longitudinal studies are needed to explore the differences between the clinical, basic, and social domains. The health management of medical researchers is crucial to promoting medical research. Additionally, policies that consider evaluating medical research and researchers are required.

## Abbreviations

aOR: adjusted odds ratio
CI: confidence interval
IF: impact factor
WHO-5: World Health Organization-Five Well-Being Index

## Supplementary information

**Additional file 1:** **Appendix Table. 1** Association between individual attitudes toward research evaluation and lower psychological well-being (Crude model). **Appendix Table. 2** Association between individual attitudes toward research evaluation and lower psychological well-being by gender (Crude model). **Appendix Table. 3** Association between individual attitudes toward research evaluation and lower psychological well-being by job rank (Crude model). **Appendix Table. 4** Lower psychological well-being of researchers by affiliated institution.

## Declarations

### Availability of data and materials

All data are incorporated into the article. AM is the guarantor of this study, has full access to all the data in this study, and takes responsibility for the integrity of the data and the accuracy of the data analysis.
